# Proteomic analysis of synovial fluid from rheumatic arthritis and spondyloarthritis patients

**DOI:** 10.1186/s12014-020-09292-9

**Published:** 2020-08-06

**Authors:** Svend Birkelund, Tue Bjerg Bennike, Kenneth Kastaniegaard, Mads Lausen, Thomas Bouet Guldbæk Poulsen, Tue Wenzel Kragstrup, Bent Winding Deleuran, Gunna Christiansen, Allan Stensballe

**Affiliations:** 1 Department of Health Science and Technology, Aalborg University, 9200 Aalborg Ø, Denmark; 2 Biogenity, 9200 Aalborg Ø, Denmark; 3 Department of Biomedicine, Aarhus University, 8000 Aarhus C, Denmark; 4 Department of Rheumatology, Aarhus University Hospital, 8000 Aarhus C, Denmark; 5 Department of Health Science and Technology, Medical Microbiology and Immunology, Aalborg University, Fredriks Bajers Vej 3b, 9200 Aalborg Ø, Denmark

**Keywords:** Proteomics, Synovial fluid, Rheumatoid arthritis, Spondyloarthritis, Cell-free DNA, Neutrophil extracellular traps

## Abstract

**Background:**

The aetiologies and pathogeneses of the joint diseases rheumatoid arthritis (RA) and spondyloarthritis (SpA) are still not fully elucidated. To increase our understanding of the molecular pathogenesis, we analysed the protein composition of synovial fluid (SF) from rheumatoid arthritis (RA) and spondyloarthritis (SpA) patients.

**Methods:**

Fifty-six synovial fluid samples (RA, n = 32; SpA, n = 24) were digested with trypsin, and the resulting peptides were separated by liquid chromatography and analysed by tandem mass spectrometry. Additionally, the concentration of cell-free DNA (cfDNA) in the synovial fluid was measured, and plasma C-reactive protein (CRP) was determined.

**Results:**

Three hundred thirty five proteins were identified within the SF. The more abundant proteins seen in RA SF were inflammatory proteins, including proteins originating from neutrophil granulocytes, while SpA SF had less inflammatory proteins and a higher concentration of haptoglobin. The concentration of cell-free DNA in the SF increased together with proteins that may have originated from neutrophils. Plasma CRP levels in both RA and SpA, correlated to other acute phase reactants.

**Conclusions:**

The proteomic results underline that neutrophils are central in the RA pathology but not in SpA, and even though inhibitors of neutrophils (migration, proteinase inhibitors) were present in the SF it was not sufficient to interrupt the disease process.

## Background

The rheumatic diseases constitute a group of diseases that affects joints, ligaments, tendons, bones and can also show systemic manifestations. Rheumatoid arthritis (RA) and spondyloarthritis (SpA) are common inflammatory systemic joint diseases, with a prevalence of 0.5–1% and 0.1–0.3% respectively [1, 2]. RA is characterized by autoantibodies, including antibodies to citrullinated proteins (adaptive immune system) and neutrophil infiltration (innate immune system) of the synovial fluid (SF), whereas SpA is an autoinflammatory disorder of the innate immune system [3]. RA is twice as common in women, while SpA is twice as common in males [1, 4]. RA typically affects the small joints of the extremities and as the disease progress, cartilage and bone destruction can occur. In SpA, arthritis often affects the small joints of the spine, sacroiliac joints, and large joints of the extremities. As both diseases progress, cartilage and bone destruction often occur. Extra articular manifestations can include enthesitis, psoriasis, uveitis, and inflammatory bowel disease.

Diagnosis of RA and SpA is based on the clinical manifestations, genetic- and biochemical markers, accompanied by imaging techniques (radiographs and magnetic resonance imaging)[5, 6]. The two main serological tests for the RA diagnosis are rheumatoid factor (RF) [7], and anti-citrullinated protein antibodies (ACPA) [8]. Citrullination is a deamination of the side chain of the amino acid arginine catalysed by citrullinating enzymes, peptidyl arginine deiminases (PAD), in particular PAD4 and PAD2, of which single nucleotide polymorphisms in PAD4 are associated with RA susceptibility [9]. In RA both RF and ACPA are positively associated with the development of a more severe disease progression [10].

In SpA, MHC class I type HLA-B27 is present in up to 90% of the patients [11] and with less than 5% of the patients being RF or ACPA positive [12]. The two diseases can thereby be differentiated, but in rare cases the two diseases can co-exist [13]. In both diseases C-reactive protein (CRP) can be increased during active disease. Additionally, in spite the known differences, understanding of the RA and SpA aetiologies remains incomplete.

The protein composition (Proteome) of SF has previously been investigated. By use of 2D-gel electrophoresis Noh et al. [14] analysed the proteome of SF from RA patients early and late in the disease development and compared it with serum from healthy donors. They found several low molecular weight proteins in the SF compared with serum. In addition, they showed that tumour necrosis-alpha-induced Adipose-Related Protein and Zinc Finger Protein, ZNF658, could be detected as possible markers for RA in serum. Using LC-MS/MS based proteomics Mateos et al. [15] pooled SF from 20 RA patients and compared the results to a pool of 20 SF from osteoarthrosis (OA) patients. They found proteins related to inflammation to be dominant in the RA group, and proteins involved in the formation and remodelling of the extracellular matrix in the OA group. Similar results were obtained by Balakrishnan et al. [16] who pooled 5 RA and 5 OA samples, respectively, and also found increased amounts of inflammatory markers of the S100 protein family and matrix metalloproteinases (MMP) in the RA group.

Using quantitative proteomics, Mahendran et al. [17] analysed individual SF samples from 10 RA, 10 psoriatic arthritis patients (PsA) and 10 controls. PsA is a subgroup of SpA and, compared to the controls, MMP3 was highly increased in both disease groups. Overall, the SF proteomics of PsA and SpA were highly similar.

To determine the molecular differences in SF from RA and SpA patients, we characterized the proteome of 56 individual SF samples from RA (n=32) and SpA (n=24) patients. This allowed us to avoid pooling samples and thereby reveal minor differences between the disease groups. To compare proteomic results with biochemical parameters, C-reactive protein (CRP) in plasma as acute phase marker, and cell-free DNA (cfDNA) as SF pseudo-marker for neutrophil extracellular traps (NETs) were determined [18].

## Methods

### Human subjects and biobank samples

The clinical samples were collected under the project “INflamation in ARThritis (INART), approved by The Central Denmark Region Committees on Health Research Ethics (1-10-72-291-12). All patients were >18 years of age, fulfilling the ACR/EULAR criteria for RA and ASAS criteria for SpA, respectively [6, 19]. The diagnosis was supported by determination of RF (38% positive) and ACPA (42% positive) for RA patients, and by determination of HLA-B27 for SpA patients (84% positive) determined by Department of Biochemistry at Aarhus University Hospital, Denmark.

### Sodium dodecyl sulphate-polyacrylamide gel electrophoresis of SF

SF was cleared for cellular debris by centrifugation at 600×*g* for 15 min at 20 °C, before storage at -80 °C. After thawing, the samples were centrifuged at 20,000×g for 10 min at 4 °C. The protein concentration was measured with the Bicinchonic Acid (BCA) Protein Assay (Thermo Fisher Scientific, Waltham, MA) according the manufactures instruction. Five µg protein in SDS-samples buffer (Expedeon, San Diego, CA) were separated by 12% SDS-polyacrylamide gel electrophoresis (Expedeon). As molecular weight standard, 2.5 µl Mark12^®^ (Thermo Fisher Scientific) was used. The proteins were stained with Krypton™ Fluorescent Protein Stain (Thermo Fisher Scientific) according the manufactures instruction and scanned on an Amersham Typhoon Biomolecular Imager (GE Healthcare, Chicago, IL).

### Sample preparation for proteomics

For sample preparation “filter-aided sample preparation” (FASP) was used [20, 21]. Briefly, 100 µg SF-protein was dissolved in 5% (w/v) sodium deoxycholate (SDC) in 50 mM triethylammonium bicarbonate (TEAB). The samples were heated to 90 °C for 5 min. Molecular weight cut-off Spinfilters 10 kDa (YM10; Millipore, Sigma-Aldrich, St. Louis, MO, USA) were used for buffer exchange between the different steps. The samples were reduced with 12 mM Tris(2-carboxyethyl) phosphine hydrochloride (TCEP), alkylated with 40 mM iodoacetamide (IAA) and digested with 0.4 μg sequencing grade modified trypsin (Promega, Fitchburg, Wisconsin, USA) resuspended in 0.5% SDC, 50 mM TEAB. After digestion, the peptides were collected, and acidified with 0.1% trifluoroacetic acid (TFA). The peptide product was purified using ethyl acetate extraction and the final product was dried down in a vacuum centrifuge and stored at − 80 °C. Prior to analysis, the samples were resuspended in 2% acetonitrile (ACN) and 0.1% TFA.

### Mass spectrometry-based proteomics analysis

The mass spectrometry-based analysis was performed according to Bennike et al. [20] in a randomized patient order. The protein solution was analysed on an automated LC-electrospray ionization (ESI) MS/MS system using an Ultimate 3000 UPLC system with a nanopump module. The system was coupled online to a Thermo-Electron Q Exactive Plus mass spectrometer (Thermo Scientific, Waltham, USA) with an emitter for nanospray ionization. Triplicate runs of each sample (5% of digested material) were loaded onto the C18 reversed phase column (Dionex; Acclaim PepMap100 C18, 5 μm precolumn and 50 cm Acclaim Pepmap RSLC, 75 μm ID main column, Thermo Scientific) and eluted with a linear gradient of 96% solvent A (1% formic acid) and 4% solvent B (acetonitrile)[20] which was increased to 35% solvent B on a 90 min ramp gradient. The MS was operated in data dependent acquisition (DDA) mode, selecting the 12 precursor-ions with the highest intensity for higher energy collisional dissociation (HCD) fragmentation. The raw- and processed data have been made available via ProteomeXchange with identifier PXD010723 [22].

### Proteomics data analysis

A label-free analysis of the proteomics data was performed in MaxQuant v1.6.0.1. The fragment scans were searched against a Uniprot database containing all reviewed Homo sapiens proteins.

(Uniprot reference proteome UPID5640; downloaded 08.2017). The following abundant peptide modifications were included in the analysis: carbamidomethylated cysteine residues (fixed), acetylation of N peptides from the N-terminal of proteins (variable), and oxidation of methionine (variable). The build-in MaxQuant target-decoy search strategy was applied and used to adjust the false discovery rate (FDR) of identified peptides and proteins to max 1%. The MaxQuant MaxLFQ feature, which estimates peptide and protein abundances based on normalized summed peptide precursor intensities, was applied. The resulting label free protein abundance (LFQ) data was processed in Perseus v1.6.0.2 [23]. All protein abundances were log2-transformed. Only for the unsupervised principle component analysis (PCA), did we replace (imputed) missing values with values drawn from a normal distribution to circumvent the problem that PCA cannot handle missing values [24, 25]. This was done by using standard parameters in Perseus for label-free proteomics data, to simulate signals from low-abundant proteins (width = 0.3, downshift = 1.8). Technical replicates were combined by the median, and differentially expressed proteins were identified by t-tests, corrected for multiple hypothesis testing using permutation-based false-positive control with standard parameters in Perseus (s0 = 0.1, FDR < 0.05). Protein function was analysed using Gene Ontology (GO) nomenclature from UniProt protein knowledgebase (UniProtKB) (http://www.uniprot.org) annotation and the software tool “Software tool for researching annotations of proteins” (STRAP) [26]. Unsupervised hierarchical clustering with Euclidean distance calculations was performed on z-score normalized data using standard parameters in Perseus (300 clusters, 10 iterations). Pearson’s correlation analyses of the LFQ values were performed as previously described [27]. Finally, for exploratory analyses we performed linear mixed effect models and random forest modelling using R.

### C-reactive protein

CRP was measured at the Department of Biochemistry at Aarhus University Hospital as part of routine care using a Cobas 6000 (Chemistry XPT).

### Cell-free DNA measurement

SF was thawed and centrifuged at 15,000×*g* for 15 min, diluted 1:25 in 10 mM Tris, pH 8.0 with 1 mM EDTA (TE-buffer). The Quant-iT™ PicoGreen™ dsDNA Assay Kit (ThermoFisher Scientific) was used according to the manufacture’s instruction using 96 well Microplates, PP, F-Bottom black chimney well design (Sigma Aldrich). Fourfold dilution series of DNA were included on all plates (1 µg/ml, 250 ng/ml, 62 ng/ml, 15.6 ng/ml, 3.9 ng/ml, 970 pg/ml, 243 pg/ml, 0 pg/ml). Samples and standards were prepared and measured in duplicates. Plates were measured on an Enspire Multimode Plate Reader (Perkin Elmer, Waltham, MA) with excitation 480 nm and emission 520 nm.

## Results

### Patient material

SF was obtained from patients visiting the outpatient clinic at Aarhus University Hospital at the time when therapeutic arthrocentesis was performed. Of the 32 RA patients, five were in treatment with TNF inhibitors (Adalimumab^®^, Certolizumab^®^ or Etanercept^®^), four with the IL-6 receptor antagonist Tocilizumab^®^ and three with T-cell activation inhibitor Abatacept^®^. Nineteen of the RA patients were in methotrexate treatment. Of the 24 SpA patients 6 were in treatment with TNF inhibitors and one with Abatacept^®^. In the RA group 38% was positive for RF and 42% positive for ACPA. In the group of SpA patients 84% was of the HLA-B27 tissue type.

### SDS-PAGE analysis of SF

**Fig. 1:**
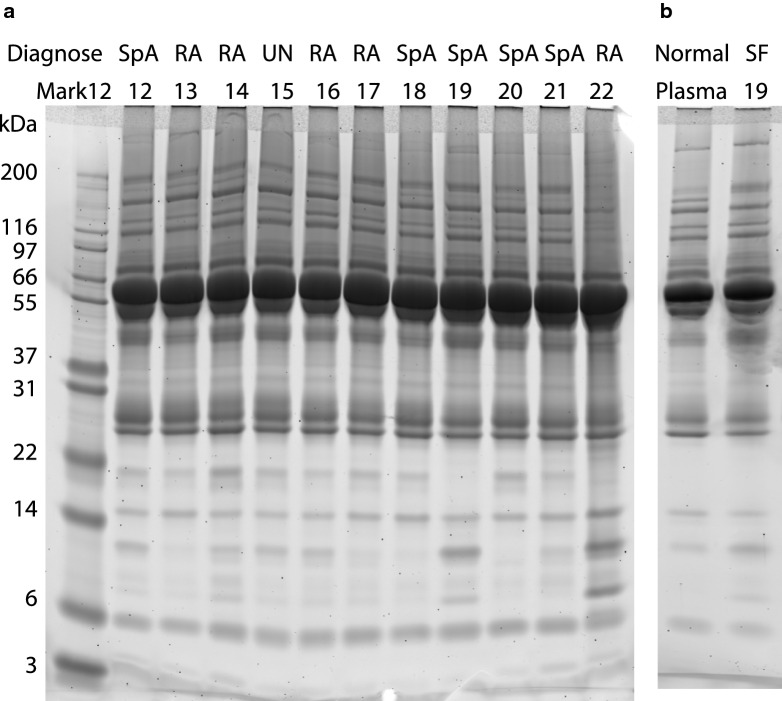
12% SDS-PAGE of SF and plasma stained with Krypton™ Fluorescent Protein Stain. **a** Lane 1: Molecular weight standard. Lanes 2–12: SF from patient 12–22. The diagnosis is marked over the patient numbers, SpA and RA. **b** Comparison of normal plasma with

The protein concentration in the 56 SF-samples varied from 15.7 to 55.4 mg/ml with a mean of 38.1 mg/ml, which is slightly higher than the ~ 25 mg/ml reported in SF from healthy persons. The higher protein-concentration is likely caused by the inflammation [28]. To analyse for major differences in the protein composition, all SF-samples were visualized by 12% SDS-PAGE (Fig. 1a, patient number 12–22). Human albumin bands with a size of 66.5 kDa were seen in all samples with a similar intensity, showing that the adjusted protein load was identical for all samples. Variation was only seen in band patterns below 20 kDa. Comparing the samples, there was no observable correlation between the low molecular weight band patterns and the diagnosis. SF is a filtrate of plasma plus proteins produced locally in the joint. Therefore, we compared a SF sample from a SpA patient to plasma from a healthy participant using SDS-PAGE (Fig. 1b), and only minor differences in low molecular weight bands were seen between normal plasma and SF from SpA patient 19. This indicates that the majority of proteins in an inflamed joint is plasma-derived proteins.

### Quantitative proteomics analysis of SF proteins in RA and SpA

Because of the high similarity between the overall protein composition of RA and SpA samples, a label-free proteomics analysis was performed to get a deeper proteome coverage and obtain relative quantitative information to reveal the differences in SF of the 56 samples (RA n = 32, SpA n =24). Each SF sample was digested in solution with trypsin, separated by nanoUPLC and peptides were sequenced using tandem MS by HCD fragmentation. All samples were analysed in triplicates, resulting in 168 MS-runs. Cumulated, we identified 335 proteins (false discovery rate (FDR) < 1%). Following stringent filtering to ensure high-quality quantitative data, 266 proteins were quantifiable cumulated in the SF samples (Additional file 1: Table S1).

### Overall SF proteome segregation by gene ontology characterization

**Fig. 2:**
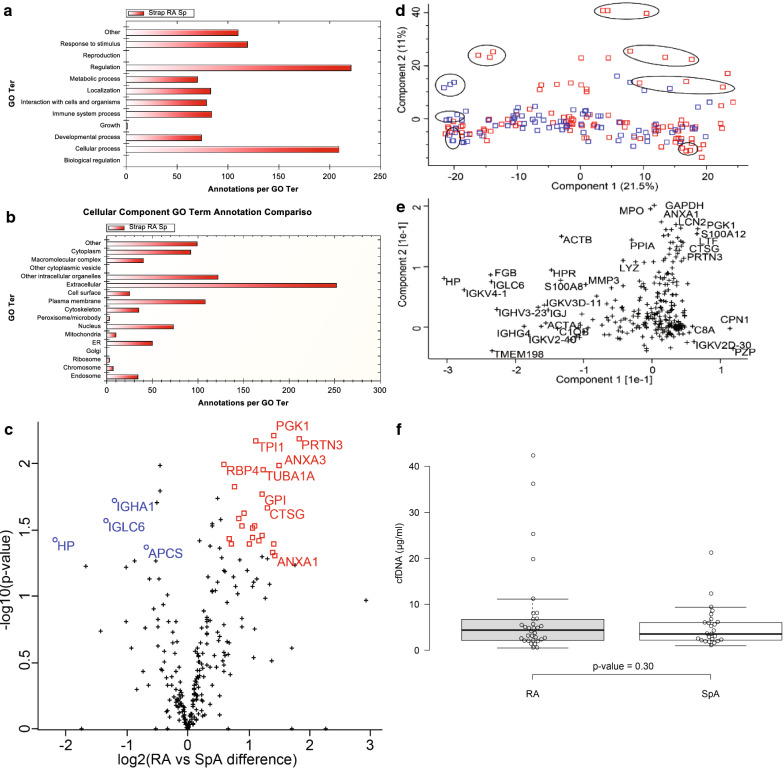
Proteomic analysis. Gene ontology for the 266 quantified proteins visualized with the STRAP software; **a** Biological processes **b** Cellular component. **c**, **d** PCA analysis of identified proteins. **d** Grouping of technical repeats (encircled) are for the majority located together. RA patients are marked red and SpA patients blue. Principle component 2 mainly separates the RA and SpA patients. **d** Analysis of proteins that contribute to 1st and 2nd components of PCA. Principal component 1 separates samples based on haptoglobin (HP), Fibrinogen beta chain (FGB) and several immunoglobulins (IG) proteins. Principal component 2 separates samples based on inflammatory proteins as Myeloperoxidase (MPO), S100-A12 protein (S100A12), Lysozyme C (LYZ), Neutrophil gelatinase-associated lipocalin (LCN2) and Cathepsin G (GTSG). E) Comparison of RA and SpA protein abundances. Gene names are given for a subset of proteins passing p-value < 0.05 as determined by t-test. □: more abundant in RA, ○: more abundant SpA, +: not significantly changed. **f** Student-T test of cfDNA concentrations in SF from RA and SpA patients. No significant difference was observed (p-value < 0.05)

The quantifiable proteins were classified according to their functional Gene ontology (GO) information obtained from UniProtKB and GO annotation and visualized with the STRAP software according to “biological process” and “cellular component” (Fig. 2). As shown in Fig. 2a (biological processes), characterization of the biological process (Fig. 2a) identified 85 proteins associated with the immune system, and in addition the groups “regulation” and “cellular process” were dominant. The GO cellular component (Fig. 2b) showed that 252 proteins were classified as “extracellular” in agreement with SF being an extracellular fluid. In addition, many proteins have the annotation “Other intracellular organelles”. This GO nomenclature covers the secretory pathway group. Furthermore, few proteins are seen in the groups: “nucleus”, “cytoplasm” and “cytoskeleton”, supporting a high degree of cellular infiltration in SF from these patients.

### Comparison of RA and SpA proteomes by Principal component analysis (PCA)

The overall similarities between the 168 LC–MS runs were investigated by an unsupervised PCA plot on the complete data set before merging the technical triplicates. The scores plot revealed that the technical repeats mostly cluster together (Fig. 2c, encircled). This demonstrates a smaller technical variance than interpersonal difference between the samples, as expected for a sensitive and robust analysis method. The first principal component (the largest possible variation 21%, X-axis) does not discriminate between RA and SpA patients (Fig 2c), whereas the second component (highest variance to first component 11%, Y-axis) is discriminative for some of the RA patients (Fig 2c).

Proteins that contribute the most to the variation in component one and two were analysed by a factor loading plot (Fig. 2d). Principle component 1 was largely separated based on haptoglobin (HP), Fibrinogen beta chain (FGB) and several immunoglobulin proteins encoded from variable (V) gene segments of both heavy chain and light chain. Principle component 2 became separated on basis of inflammatory proteins, including Myeloperoxidase, S100-A12 protein, Lysozyme C, Cathepsin G and Neutrophil gelatinase-associated lipocalin. (Fig. 2d). This indicates that the PC2 separation is based on the inflammatory joint status of the patients. Accordingly, the four RA patients with the highest PC2-value (pt. 22, 25, 34, and 64) had significantly increased SF IgM (mean increase 6.34, p-value 0.04). In these patients CRP was also increased, although not significantly.

### Analysis of SF protein differences between RA and SpA

Comparing all identified proteins of RA to all of SpA using t-tests, none of the proteins passed multiple hypothesis correction (q-value < 0.05). However, applying a less strict cut-off without multiple hypothesis correction (p-value < 0.05, log2 (fold change) > 0.5 or < − 0.5), 25 more abundant proteins in RA compared to SpA were identified, in addition to 4 less abundant proteins (Fig. 2e, Table 1). The majority of the more abundant RA-proteins are present in neutrophil granulocytes and monocytes (Table 1 marked ^a^) or proteins involved in the glycolysis (Table 1 marked ^b^). This is in agreement with the preliminary conclusions from the PCA, and indicates the presence of infiltrating neutrophils in the SF from RA patients, in agreement with previous reports [3]. In addition, the data demonstrates the significantly lower/lack of infiltrating neutrophils in SpA, highlighting a central difference in the pathogenesis of RA and SpA.

**Table 1:** Proteins with a significant abundance difference (p-value < 0.05, log2 (fold change) > ± 0.5) between RA and SpA synovial fluid

RA SpA fold change (%)	p-value	Protein names
357	0.006	Myeloblastin^a^
281	0.010	Annexin A3
270	0.050	Annexin A1
267	0.006	Phosphoglycerate kinase 1
267	0.041	Alpha-actinin-1
263	0.047	Myeloid cell nuclear differentiation antigen^a^
247	0.022	Cathepsin G^a^
236	0.011	Tubulin alpha-1A chain
234	0.017	Glucose-6-phosphate isomerase^b^
232	0.035	Ras-related C3 botulinum toxin substrate 2
224	0.039	Heat shock-related 70 kDa protein 2
217	0.007	Triosephosphate isomerase^b^
213	0.030	Moesin
209	0.031	Pyruvate kinase^b^
208	0.036	Adenylyl cyclase-associated protein 1^a^
202	0.040	Glyceraldehyde-3-phosphate dehydrogenase^b^
189	0.024	14-3-3 protein zeta/delta
186	0.030	Beta-2-microglobulin
179	0.026	Profilin-1
171	0.015	Phosphoglycerate mutase 1^b^
164	0.041	Plastin-2
160	0.037	Transgelin-2
151	0.010	Retinol-binding protein 4
145	0.026	Complement factor D
144	0.044	Transketolase
62	0.043	Serum amyloid P-component
43	0.019	Ig alpha-1 chain C region
40	0.027	Ig lambda-6 chain C region
22	0.038	Haptoglobin

^a^Proteins specific for neutrophile grunolocytes and monocytes

^b^Enzymes involved in glycolysis

### Correlation of protein changes to concentration of cell-free DNA in SF

Cell-free DNA (cfDNA) in plasma is an inflammatory marker for RA and has been proposed as a predictive marker for biological disease-modifying anti-rheumatic drugs (DMARD) treatment [29, 30]. cfDNA in the SF was measured by a fluorometric method. The SF cfDNA concentration was found to vary between 0.5 to 42.2 µg/ml. These values are an order of tree magnitude higher than can be observed in plasma from RA patients [29, 30], and it reflects that cfDNA originates from cells present in SF. SF cfDNA could be measured in both patient groups, but no statistically significant difference between the intra articular cfDNA concentrations was observed between the RA and SpA patients (RA_mean_ = 7.3 µg/ml, SpA_mean_ = 5.1 µg/ml, t-test p-value = 0.30) (Fig. 2f). The SF samples were centrifuged to remove cells before freezing and to avoid the release of cellular DNA. However, it cannot be excluded that cell lysis occurred after collection and prior to centrifugation. With this reservation in mind, the SF levels of cfDNA was correlated to the SF protein abundance levels to identify functional proteins, which could elucidate the biological interpretation of the cfDNA measurements. Sixty-eight proteins correlated significantly (p-value < 0.05) with the amount of cfDNA in the SF (Table 2).

**Table 2:** Correlation Analysis: Synovial fluid proteins identified by mass spectrometry across all RA and SpA samples correlating significantly (p-value < 0.05) to synovial fluid cfDNA

UPID	Protein name	Gene name	R	p-value	NETs
P62805	Histone H4	HIST1H4A	0.9170	1.52E−06	+
P14780	Matrix metalloproteinase-9	MMP9	0.8141	4.02E−06	+
P80188	Neutrophil gelatinase-associated lipocalin	LCN2	0.7925	3.88E−08	+
P07900	Heat shock protein HSP 90-alpha	HSP90AA1	0.7314	4.40E−06	
P37837	Transaldolase	TALDO1	0.7292	7.12E−03	
P02788	Lactotransferrin	LTF	0.7158	5.62E−10	+
P20160	Azurocidin	AZU1	0.7114	4.61E−05	+
Q99880	Histone H2B type 1-L	HIST1H2BL	0.7008	1.61E−05	+
P12814	Alpha-actinin-1	ACTN1	0.6964	3.86E−05	+
P61626	Lysozyme C	LYZ	0.6946	1.57E−08	+
P24158	Myeloblastin	PRTN3	0.6803	4.89E−05	+
P15153	Ras-related C3 botulinum toxin substrate 2	RAC2	0.6803	1.31E−04	
P52566	Rho GDP-dissociation inhibitor 2	ARHGDIB	0.6694	1.25E−03	
P08246	Neutrophil elastase	ELANE	0.6660	1.35E−03	+
P05164	Myeloperoxidase	MPO	0.6584	4.89E−07	+
P52209	6-phosphogluconate dehydrogenase, decarboxylating	PGD	0.6529	4.04E−04	
P0DMV9	Heat shock 70 kDa protein 1B	HSPA1B	0.6524	1.35E−03	
P63261	Actin, cytoplasmic 2	ACTG1	0.6419	1.28E−07	
P00558	Phosphoglycerate kinase 1	PGK1	0.6270	3.28E−05	
Q99878	Histone H2A type 1-J	HIST1H2AJ	0.6265	1.05E−03	+
P04406	Glyceraldehyde-3-phosphate dehydrogenase	GAPDH	0.6096	3.33E−06	
P06744	Glucose-6-phosphate isomerase	GPI	0.6007	3.99E−03	
P12429	Annexin A3	ANXA3	0.5955	2.14E−03	
P08311	Cathepsin G	CTSG	0.5944	1.66E−04	+
P06733	Alpha-enolase	ENO1	0.5942	1.38E−06	+
P14618	Pyruvate kinase PKM	PKM	0.5898	2.15E−06	
P06702	Protein S100-A9	S100A9	0.5857	2.12E−06	+
P07737	Profilin-1	PFN1	0.5778	5.90E−06	
P08670	Vimentin	VIM	0.5745	1.58E−05	
P60174	Triosephosphate isomerase	TPI1	0.5720	4.09E−04	
P13796	Plastin-2	LCP1	0.5704	5.45E−06	+
Q01518	Adenylyl cyclase-associated protein 1	CAP1	0.5698	1.53E−04	
P09211	Glutathione S-transferase P	GSTP1	0.5578	1.67E−03	
P00338	l-Lactate dehydrogenase A chain	LDHA	0.5531	4.14E−03	
P04083	Annexin A1	ANXA1	0.5486	1.40E−04	
P01033	Metalloproteinase inhibitor 1	TIMP1	0.5463	3.21E−04	
P26038	Moesin	MSN	0.5314	4.19E−04	
P62937	Peptidyl-prolyl cis-trans isomerase A	PPIA	0.5142	1.85E−04	
P30740	Leukocyte elastase inhibitor	SERPINB1	0.5103	4.34E−02	
P18669	Phosphoglycerate mutase 1	PGAM1	0.5080	3.13E−04	
Q71U36	Tubulin alpha-1A chain	TUBA1A	0.4917	1.07E−02	
P22894	Neutrophil collagenase	MMP8	0.4728	4.74E−03	
P80511	Protein S100-A12	S100A12	0.4689	5.16E−03	
P63104	14-3-3 protein zeta/delta	YWHAZ	0.4675	2.70E−03	
P29401	Transketolase	TKT	0.4575	1.58E−03	
P09960	Leukotriene A-4 hydrolase	LTA4H	0.4572	3.91E−03	
P23528	Cofilin-1	CFL1	0.4462	1.89E−03	
P68133	Actin, alpha skeletal muscle	ACTA1	0.4370	2.01E−02	
P08133	Annexin A6	ANXA6	0.4199	7.79E−03	
P02679	Fibrinogen gamma chain	FGG	0.4183	1.34E−03	
P36222	Chitinase-3-like protein 1	CHI3L1	0.4106	7.67E−03	
P12111	Collagen alpha-3(VI) chain	COL6A3	0.3898	3.26E−03	
P07195	l-Lactate dehydrogenase B chain	LDHB	0.3686	3.19E−02	
P04075	Fructose-bisphosphate aldolase A	ALDOA	0.3684	2.94E−02	
P02741	C-reactive protein	CRP	0.3470	8.78E−03	
P04003	C4b-binding protein alpha chain	C4BPA	0.3119	2.17E−02	
P02763	Alpha-1-acid glycoprotein 1	ORM1	0.3061	2.18E−02	
P02750	Leucine-rich alpha-2-glycoprotein	LRG1	0.3046	2.24E−02	
P04040	Catalase	CAT	0.2955	2.70E−02	
P07225	Vitamin K-dependent protein S	PROS1	0.2941	2.78E−02	
P08603	Complement factor H	CFH	0.2883	3.12E−02	
P0DJI8	Serum amyloid A-1 protein	SAA1	0.2866	3.56E−02	
P60709	Actin, cytoplasmic 1	ACTB	0.2793	4.09E−02	
P49747	Cartilage oligomeric matrix protein	COMP	− 0.2706	4.37E−02	
P02751	Fibronectin	FN1	− 0.2939	2.79E−02	
Q13790	Apolipoprotein F	APOF	− 0.4627	3.02E−03	
Q96RL7	Vacuolar protein sorting-associated protein 13A	VPS13A	− 0.5619	1.23E−02	
Q66K66	Transmembrane protein 198	TMEM198	− 0.5646	2.27E−02	

### Correlation of proteins found changed/increased by proteomic in SF to regulatory pathways

To identify underlying biological themes and pathways of the correlating proteins, a GO enrichment analysis using the Reactome pathway database, calculated with all identified SF-proteins as background was performed [31]. The list of synovial fluid proteins with a positive correlation with cfDNA, was significantly enriched (FDR < 0.05) in five biological pathways (Table 3). Thirty-nine of the proteins were tagged as “Immune System” (R-HSA-168256), 31 as “Innate Immune System” (R-HSA-168249) and 26 of the 68 proteins (38%) were categorized as “Neutrophil degranulation” (R-HAS-6798695). The Glycolysis (R-HAS-70171) pathway was also significant enriched, in agreement with the anaerobic metabolism of neutrophils and liberation of the cytoplasmic enzymes upon cell disruption [32].

**Table 3:** Statistical significant enriched Reactome biological pathways for synovial fluid proteins associated with the synovial fluid cfDNA concentration

Reactome ID	Name	#Proteins	FDR
R-HSA-70171	Glycolysis	8	1.15e−02
R-HSA-6798695	Neutrophil degranulation	26	1.15e−02
R-HSA-71387	Metabolism of carbohydrates	11	3.36e−02
R-HSA-70263	Gluconeogenesis	7	4.85e−02
R-HSA-168256	Immune system	39	4.85e−02

NETs are formed in the process of NETosis where neutrophils ejects DNA with histones, in addition to cytoplasmic and secretory granules to form a web-like structure [33, 34]. Of the 11 most correlated proteins to cfDNA, 9 were known NETs proteins (Table 2, labelled “+”). Therefore, the presence of cfDNA correlates with proteins predominantly found in neutrophil granulocytes in both RA and SpA SF, and in vivo liberation of DNA prior to sample collection is therefore likely and not a sampling artefact.

**Fig. 3:**
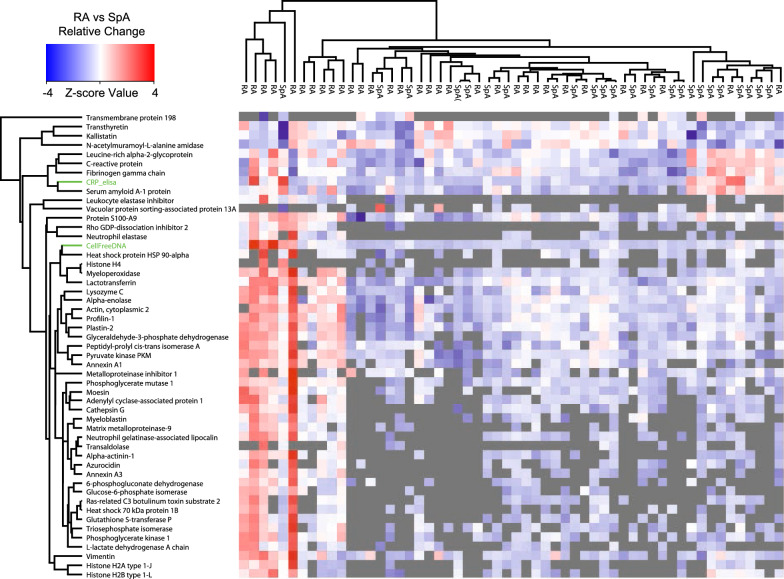
Hierarchical clustering of cfDNA and plasm CRP correlated proteins. For the clustering all proteins correlating positive R > 0.5 or negativ R < − 0.5 to plasma CRP or cfDNA were selected (Tables 2, 4). Values of plasma CRP (log2) and cfDNA (log2) were included in the clustering (labelled green). The diagnosis is marked over the clustering. NGAL is the abbreviation for Neutrophil gelatinase-associated lipocalin

Twenty-nine proteins known to be associated with NETs formation have been identified by MS [34, 35]. Of these 29 proteins, 21 were identified in the SF. To visualize the cfDNA correlation to NETs proteins a hierarchical clustering with the 21 proteins and SF cfDNA was performed (Fig. 3). The analysis showed that high concentration of cfDNA was correlated to presence of NETs proteins. NETs formation is enhanced by Resistin, that can be produced by synoviocytes in joints of RA patients [36, 37]. The receptor for Resistin is the Adenylyl cyclase-associated protein 1 (CAP1) [36]. Accordingly, CAP1 was found to be more abundant in the RA group (Table 1) and correlated significantly and positively with cfDNA (R = 0.5698, p-value = 1.53*10^−4^) (Table 2). The findings indicate that NETs formation is more abundant in RA-joints compared to SpA, again pointing to the involvement of NETs in RA which recently has been demonstrated to sustain inflammation in other inflammatory diseases.

### Correlation of plasma CRP to SF proteins

A common inflammatory marker used in SpA and RA is plasma-CRP, which has a half-life of 19 h [38]. Therefore, in addition to cfDNA, the measured plasma-CRP was correlated to protein abundances in SF, with the aim to identify inflammation-associated proteins. Forty-one proteins were found to correlate significantly with plasma-CRP (p-value < 0.05), 22 of which correlated positively and 19 negatively. Reassuringly, SF-CRP correlated positively with plasma-CRP (R = 0.7148, p-value = 6.12*10^−10^), verifying the validity of the MS-based proteomics data (Table 4). The other proteins correlating positively to plasma-CRP were also acute phase reactants, e.g. alpha-1-antichymotrypsin, serum amyloid A-1 protein, and, further, other protein groups influenced by plasma-CRP such as complement system proteins (Table 4). Proteins correlating negatively included known “Negative” acute-phase proteins such as albumin, transferrin, transthyretin and retinol-binding protein 4 [39, 40].

**Table 4:** SF proteins identified by proteomics with statistically significant correlation (p-value < 0.05) to plasma CRP

UPID	Protein name	Gene name	R	p value
P0DJI8	Serum amyloid A-1 protein*	SAA1	0.7254	5.47E−10
P02741	C-reactive protein*	CRP	0.7148	6.12E−10
P02750	Leucine-rich alpha-2-glycoprotein*	LRG1	0.5769	3.26E−06
P02679	Fibrinogen gamma chain*	FGG	0.5399	1.75E−05
Q7Z4H8	KDEL motif-containing protein 2	KDELC2	0.4856	4.82E−02
P18428	Lipopolysaccharide-binding protein	LBP	0.4765	2.05E−04
P07225	Vitamin K-dependent protein S*	PROS1	0.4328	8.64E−04
Q06033	Inter-alpha-trypsin inhibitor heavy chain H3	ITIH3	0.4321	9.85E−04
P00751	Complement factor B	CFB	0.4182	1.34E−03
P02671	Fibrinogen alpha chain	FGA	0.3954	2.56E−03
P06702	Protein S100-A9*	S100A9	0.3913	2.86E−03
P02748	Complement component C9	C9	0.3893	3.02E−03
P01011	Alpha-1-antichymotrypsin	SERPINA3	0.3868	3.23E−03
P01009	Alpha-1-antitrypsin	SERPINA1	0.3865	3.26E−03
P68133	Actin, alpha skeletal muscle*	ACTA1	0.3791	4.66E−02
P02743	Serum amyloid P-component	APCS	0.3657	6.03E−03
P08603	Complement factor H*	CFH	0.3298	1.30E−02
P36222	Chitinase-3-like protein 1*	CHI3L1	0.3170	4.34E−02
P02788	Lactotransferrin*	LTF	0.3042	2.26E−02
P05156	Complement factor I	CFI	0.2866	3.23E−02
P04003	C4b-binding protein alpha chain*	C4BPA	0.2727	4.60E−02
Q14624	Inter-alpha-trypsin inhibitor heavy chain H4	ITIH4	0.2683	4.56E−02
P27169	Serum paraoxonase/arylesterase 1	PON1	− 0.2719	4.27E−02
P02753	Retinol-binding protein 4	RBP4	− 0.2900	3.02E−02
P05452	Tetranectin	CLEC3B	− 0.2923	2.88E−02
P02656	Apolipoprotein C-III	APOC3	− 0.2934	2.82E−02
Q92954	Proteoglycan 4	PRG4	− 0.2970	2.62E−02
P01871	Ig mu chain C region	IGHM	− 0.2997	2.48E−02
P19823	Inter-alpha-trypsin inhibitor heavy chain H2	ITIH2	− 0.3006	2.44E−02
P19827	Inter-alpha-trypsin inhibitor heavy chain H1	ITIH1	− 0.3293	1.32E−02
P02751	Fibronectin*	FN1	− 0.3301	1.30E−02
P22352	Glutathione peroxidase 3	GPX3	− 0.3325	2.74E−02
P02647	Apolipoprotein A-I	APOA1	− 0.3343	1.18E−02
P43652	Afamin	AFM	− 0.3650	5.67E−03
P02787	Transferrin	TF	− 0.3669	5.41E−03
P05154	Plasma serine protease inhibitor	SERPINA5	− 0.3706	9.51E−03
P06396	Gelsolin	GSN	− 0.3730	4.63E−03
P02768	Albumin	ALB	− 0.4892	1.30E−04
Q96PD5	*N*-Acetylmuramoyl-L-alanine amidase	PGLYRP2	− 0.5021	8.06E−05
P02766	Transthyretin	TTR	− 0.5277	2.93E−05
P29622	Kallistatin	SERPINA4	− 0.5492	1.17E−05

Proteins marked with * are also positive correlated to SF cfDNA

### Correlation of SF proteins to plasma-CRP and SF cfDNA

The two inflammatory markers, plasma-CRP and SF cfDNA correlated, but not strongly (R = 0.4765, p-value = 2.01*10^−4^). However, the proteins correlating positively or negatively to each of the two markers were considerably different, and of the 109 statistically significantly correlating proteins, only 12 (11%) were common (Tables 2, 4). Hierarchical clustering with positive correlated proteins to both cfDNA (Fig. 3) and positive as well as negative correlated proteins to plasma CRP, showed a distinct clustering (Fig. 3) with a group of 11, predominantly RA patients with NETs markers (Fig. 3, left). Three of these patients were in biological treatment, showing that the treatment was not responsible for the generation of cfDNA. A group of 10, predominantly SpA patients, correlated with acute phase reactants without NETs proteins (Fig. 3, right), demonstrating the involvement of NETs in RA and the likely subgrouping of the SpA patients. On average a stronger correlating with cfDNA to granulocyte proteins (mean absolute difference 0.13, p-value 1.15*10^−6^) were seen than to plasma CRP.

## Discussion

The findings presented in the present study is, to our knowledge, the first unbiased proteomic approach of examining and comparing RA and SpA SF protein composition. The findings were further correlated with plasma CRP levels and SF cfDNA for discriminating factors. Among the RA patients, proteins from neutrophils were more dominant in SF. Haptoglobin was the only protein found reduced in SF from RA compared with SpA patients, in accordance with higher levels of the scavenging receptor for haptoglobin–haemoglobin CD163 in RA patients [41]. Proteins from neutrophils and glycolytic enzymes correlated strongly to cfDNA in SF predominantly from RA patients. CRP and other acute phase reactants were seen in both RA and SpA patients, but high amounts of acute phase reactants were also detected in SpA patients without neutrophil granulocyte markers.

**Fig. 4:**
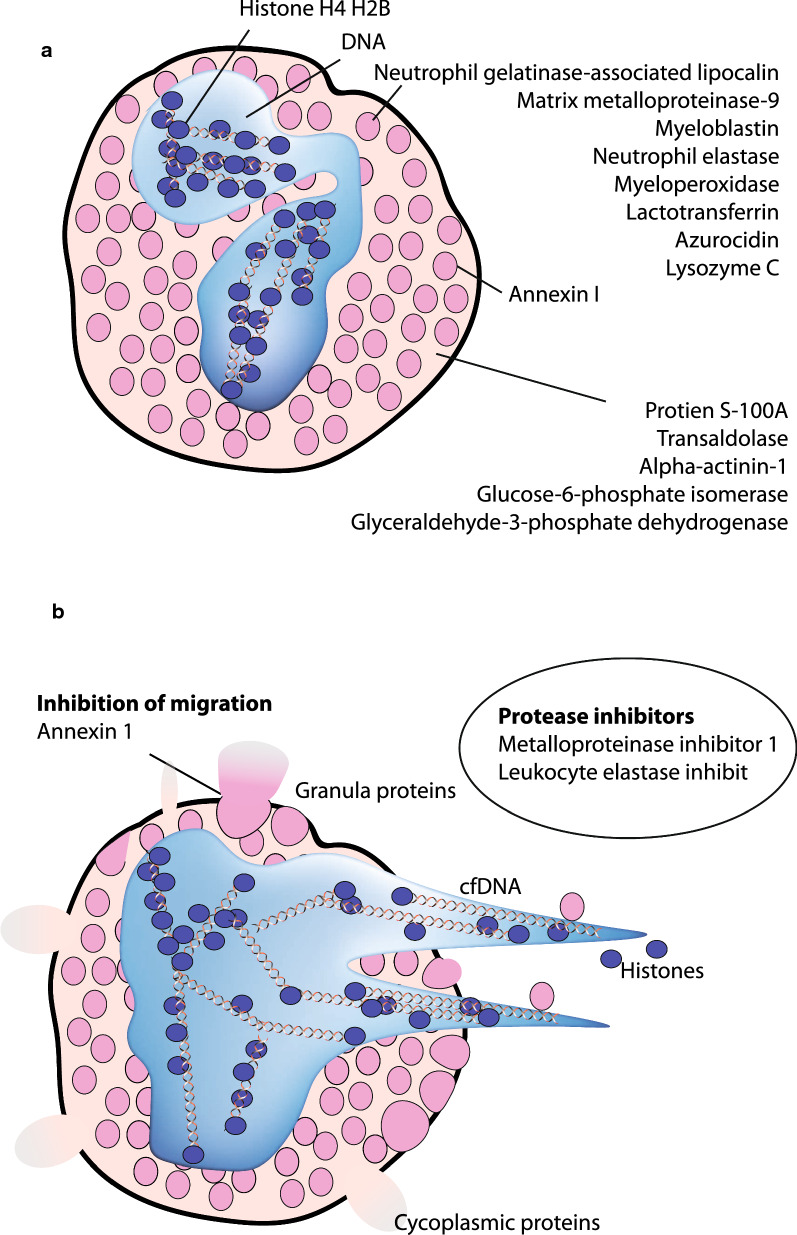
Localization of neutrophil proteins that correlate to cfDNA detected in SF. **a** Schematic drawing of a neutrophil and localisation of proteins correlating to cfDNA. Nucleus (blue) shows DNA and chromatin. Cytoplasmic granules are shown in pink. Annexin 1 is present in the granular membranes but translocated to the cytoplasmic membrane upon degranulation/NETs formation. Cytoplasm (light pink). **b** During NETosis proteins from all compartments can be released. In inflamed RA joint—both granular, cytoplasmic, nuclear proteins, Annexin 1 and cfDNA were detected in SF. Annexin 1 is an inhibitor of migration of neutrophil to inflammation. Metalloproteinase inhibitor 1 and Leukocyte elastase inhibitor were correlated to cfDNA. The origin of these inhibitors is unknown

NETs is formed by neutrophils undergoing NETosis, where DNA is expelled from the cells within the tissue lining and in biofluids to form web-like structures together with histones, S100-proteins, and proteins stored in secretory granules (Fig. 4) [34]. NETs markers, as circulating cfDNA, can be used to monitor treatment efficiency of biological DMARD in RA patients [29].

Correlation of SF cfDNA to known NETs proteins in SF was found, indicating that the origin of cfDNA was from neutrophils and thereby that NETosis likely had occurred in the joints in vivo. Due to the presence of both the cytoplasmic S100-proteins and the nuclear histone proteins, degranulation of neutrophils without NETosis is unlikely. The DNA-binding protein, Histone H4, is the most correlated protein with cfDNA. Furthermore, MMP9, NGAL (Neutrophil gelatinase-associated lipocalin) and other NETs proteins were also strongly correlated to the presence of cfDNA in the samples (Table 2), indicating that the cfDNA originated from NETosis process. Cytoplasmic proteins as Glucose-6-phosphate isomerase and Glyceraldehyde-3-phosphate dehydrogenase correlated also to cfDNA indicating cell rupture (Fig. 4).

Neutrophils contain many proteases with tissue destructive effects. In the SF with high cfDNA, MMP9, MMP8, neutrophil elastase, Myeloblastin and Cathepsin G, that can contribute to destruction, were found (Table 2). MMP9 forms a high molecular weight complex with NGAL, that protects MMP9 from degradation and thereby prolongs its proteolytic activity and tissue destruction [42]. NGAL is upregulated in human neutrophils by granulocyte-macrophage colony-stimulating (GM-CSF), and in SF of RA compared to OA patients, higher concentrations of NGAL was measured [43]. This is in agreement with reports of higher plasma MMP9 in RA patients compared with OA, and that a higher enzymatic activity was found in SF of RA patients [44, 45]. Furthermore, neutrophils from RA patients compared to healthy controls have a tendency to undergo NETosis easier when treated with PMA [18]. MMP9 could also be produced by synoviocytes in SF from RA patients, but its high correlation to cfDNA shows that the MMP9 most likely originated from neutrophils [46].

Metalloproteinase inhibitor 1 and Leukocyte elastase inhibitor both correlated to cfDNA, but the cell type producing these inhibitors are unclear (Fig. 4). Metalloproteinase inhibitor 1 can form a complex with MMP9 and NGAL. Therefore, the in vivo MMP9 activity is difficult to determine. Ahrens et al. [45] have shown active MMP9 gelatinase activity in SF from RA patients by use of gel zymography, and thus, MMP9 was separated from Metalloproteinase inhibitor 1. Annexin 1, released by neutrophils, is an inhibitor of migration of neutrophils from the blood to the inflamed site, and even though Annexin 1 was present in the SF with cfDNA, it was unable to prevent disease progression (Fig 4).

Katano et al. [43] proposed GM-CSF as a target for treatment of RA and this is now supported by human trials. Patients with high SF cfDNA and NGAL could therefore be a distinct clinical endotype that may benefit from such a treatment [47]. Sato et al. [37] proposed that Resistin produced by synovial tissue could be important for the pathogenesis of RA. The receptor for Resistin, CAP1, was present in SF and correlated to cfDNA. CAP1 is a cytoplasmic protein, but is translocated to the cell membrane when Resistin is present [48]. Resistin enhances NETosis [36], but whether CAP1 in SF can function as a receptor antagonist in its free form is presently unknown.

Plasma CRP is a central marker used in combination with 28-joint Disease Activity Score (DAS28CRP) and Ankylosing Spondylitis Disease Activity Score (ASDAS) to determine disease progression and treatment success, that correlates to disease progression [49]. When plasma CRP was correlated to SF proteomic data, other known positively and negatively regulated acute phase reactants were found (Tables 3 and 4, Fig. 3). Proteins correlated to plasma CRP and cfDNA, respectively, had only 12% proteins in common. Leucine-rich alpha-2-glycoprotein (LRG1) is correlated to both CRP and cfDNA. It is an acute phase protein induced primarily by IL-6 in the liver [50, 51], but is also a neutrophil secondary granule protein released together with lactoferrin, and thus its correlation to both CRP and cfDNA is explainable [52]. LRG1 inhibits the anti-proliferative effect of transforming growth factor ß1 (TGFß1) on myeloid cells [51]. TGFß1 regulates the anti-inflammatory process in the synovial membrane in RA patients, and is important for the self-regulation that can result in remission periods [53]. In RA patients treated with anti-interleukin-6 receptor antibody (RoActemra) LRG1 was a better marker for remission than CRP, matrix metalloproteinase 3 level and erythrocyte sedimentation rate [54]. As shown in the present study, this can be due to LRG1 being a maker for both acute phase reactants and for neutrophil degranulation/NETosis. As shown in Fig. 3, a group of manly SpA patients had high CRP and acute phase reactants without granulocyte markers, confirming the central role of neutrophil granulocytes in the pathogenesis of RA.

The differences between RA and SpA identified here are in line with results from randomized clinical trials with already approved drugs. Thus, T-cell targeted therapies such as inhibitors of IL-17 has shown efficacy in SpA but not in RA. In contrast, therapies targeting myeloid derived cytokines such as inhibitors of IL-1 and IL-6 are effective in RA but not in SpA [55]. Our findings also support that RA is a very heterogenous disease as proposed by others [56]. Thus, a subgroup of RA patients with high neutrophil activation was found. High degree of neutrophil priming and NETosis is also present in other diseases such as systemic lupus erythematosus and granulomatosis with polyangiitis [33, 57]. Some of the drugs used for treatment of these diseases such as the C5a receptor inhibitor Avacopan^®^ could, therefore, also be effective in RA patients with high neutrophil activation. In this way, the study could help guide future drug development to treat immune mediated inflammatory arthritis.

## Conclusions

The proteomics of SF from SpA and RA patients showed a marked difference in the amounts of proteins from the innate immune system, primarily originating from neutrophil granulocytes. The presence of these proteins was more pronounced in the RA patient group. These proteins were also correlated to SF cfDNA indicating NETosis. Neutrophils produce IL-6 that induces acute phase reactants, but surprisingly, little correlation between NETs proteins/cfDNA and acute phase reactants/CRP was seen, indicating that two different inflammatory mechanisms are used for increase in CRP and cfDNA. This is in agreement with the recent finding that mature neutrophils are unresponsive to IL-6 due to the absence of gp130 in the IL-6 receptor complex [58]. Some of the patients with high cfDNA were in treatment with anti-interleukin-6 receptor (RoActemra^®^) or TNF-α antagonists. This may have influenced the amounts of acute phase reactants. However, in a patient material as presented in this study, SF cfDNA is an indicator of intraarticular NETosis. Therefore, SF cfDNA measurement may be used as an indicator of severe arthritis, and our findings demonstrate the involvement of NETs in RA but less in SpA pathogenesis.

## Supplementary information

**Additional file 1.** Combined list of identified proteins in all samples.

## Data Availability

The raw- and processed data have been made available via ProteomeXchange with identifier PXD010723

## Abbreviations

ACPA: anti-cyclic citrullinated protein antibodies
cfDNA: cell-free DNA
CRP: C-reactive protein
ESI: LC-electrospray ionization
FDR: false discovery rate
HCD: higher energy collisional dissociation
LFQ: label free quantification
MHC: major histocompatibility complex
OA: osteoarthrosis
PAD: peptidyl arginine deiminases
PCA: principle component analysis
RA: Rheumatoid arthritis
MMP: Matrix metalloproteinases
MS/MS: tandem mass spectrometry
NETs: neutrophil extracellular traps
RF: rheumatoid factor
SDC: sodium deoxycholate
SF: synovial fluids
SpA: spondyloarthritis
UniProtKB: UniProt protein knowledgebase
UPLC: ultra-performance liquid chromatography
TEAB: triethylammonium bicarbonate
